# 3D Printing of Cement-Based Materials Using Seawater for Simulated Marine Environments

**DOI:** 10.3390/ma19010093

**Published:** 2025-12-26

**Authors:** Fabian B. Rodriguez, Caiden Vugteveen, Xavier Fross, Hui Wei, Michael E. Himmel, Anastasia N. Aday, Drazenka Svedruzic, John T. Kevern

**Affiliations:** 1 Mechanical and Thermal Engineering Sciences Directorate, National Laboratory of the Rockies, 15013 Denver West Parkway, Golden, CO 80401, USA; fabian.rodriguez@nrel.gov (F.B.R.); ana.aday@nrel.gov (A.N.A.); 2 Bioeconomy and Sustainable Transportation Directorate, National Laboratory of the Rockies, 15013 Denver West Parkway, Golden, CO 80401, USA; hui.wei@nrel.gov (H.W.); mike.himmel@nrel.gov (M.E.H.); drazenka.svedruzic@nrel.gov (D.S.)

**Keywords:** 3D printing of concrete, marine environments, seawater, rheological properties, chemical admixtures, underwater 3D printing

## Abstract

Global demand for adaptable and rapidly deployable construction solutions in offshore, coastal, and fluvial environments continues to rise, driven by pressing needs to develop energy platforms, improve coastal resilience, and support emergency response in the face of natural disasters. Increased investment in human-made coastal infrastructure, such as piers, support structures for power lines, offshore wind farms, and seawall protection systems, further underscores this trend. This study investigates the development of printable concrete mixtures for underwater environments using seawater as a replacement for freshwater, using a 3D printing syringe-based extrusion system. The effect of seawater addition and the printing medium (in air vs. underwater) was assessed via rheological and mechanical performance characterization. The results indicate rheological properties are favorable for seawater adoption by producing mixtures with higher yield stress and viscosity with the same levels of admixtures used for freshwater. Seawater-based mixtures demonstrated superior dimensional stability compared to freshwater counterparts, maintaining cross-sectional geometry, while compressive strength results showed no statistical differences between in-air and underwater samples. However, flexural strength was significantly influenced by geometry and printing medium. These findings establish critical rheological parameters for printable underwater mixtures and highlight the need for optimized curing strategies and layer bonding techniques to improve interfacial strength in underwater 3D printing applications.

## 1. Introduction

Marine and coastal infrastructure represents a challenging yet exciting frontier in modern construction. These environments are indispensable for supporting national energy and transportation systems, from offshore petroleum platforms to global trade through ports, harbors, subsea pipelines, and coastal protection structures [1,2]. Coastal regions are also home to significant populations, with approximately 40% of the United States’ population living in counties along the east and west coasts, as reported by the National Oceanic and Atmospheric Administration (NOAA) [3]. These areas face increasing threats from flooding, hurricanes, and erosion, which underscores the urgent need for adaptable coastal infrastructure planning [4,5]. Such challenges demand significant infrastructure spending each year to ensure public safety and protect local economies with preventive measurements, in addition to the cost of recovering affected zones [6,7,8,9,10]. Beyond natural disaster resilience and energy production, marine infrastructure extends to global trade, where port and harbor expansion is essential to meet growing maritime traffic. Furthermore, marine infrastructure also plays a key role in environmental viability in the long term and biodiversity preservation such as artificial reef systems, which have attracted a growing share of public investments, aiming to mitigate coastal erosion, enhance biodiversity, and support fisheries [11].

The diversity of investments and their transformative impact highlight the critical role marine infrastructure plays in shaping the future of sustainable development, energy, and coastal resilience. Consequently, the quality and performance of construction materials used in these environments are vital to ensuring the longevity and functionality of marine structures [2,12]. Harsh conditions in marine environments require advancements in material formulations to achieve structural integrity, low porosity, and performance for design service lives of 50 years or more. The development of sustainable concrete for marine applications has increasingly focused on leveraging local resources, such as seawater and sea sand as alternatives to freshwater and mined aggregates to reduce environmental depletion and transportation costs in addition to the incorporation of supplementary cementitious materials [13,14,15,16]. However, the adoption of local materials comes with its own challenges, such as the need to customize and optimize cement formulations, potential variability in fresh concrete properties, and long-term durability concerns in aggressive marine settings as well as a satisfactory integration with marine ecosystems [17,18]. Addressing these challenges and developing strategies for effective deployment of construction materials is fundamental to enable the next generation of resilient and sustainable marine infrastructure, achieving an optimal balance between cost, performance, and resilience [19,20].

3D printing of concrete (3DPC) has emerged as a transformative, versatile, cost-effective technology gaining traction across the construction industry. While much of the spotlight has initially been on its applications in residential construction, 3DPC is proving to be a catalyst for innovation in infrastructure [21,22]. These qualities make 3DPC particularly promising for maritime and coastal applications. The potential for rapid deployment, coupled with the flexibility to tailor materials specifically for marine environments, positions 3DPC as a game-changer for addressing the growing global demand for resilient and sustainable marine infrastructure.

One exciting avenue for 3DPC in marine applications is its potential use in offshore and coastal construction systems, including artificial reefs, seawalls, underwater pipelines, and multifaceted coastal protection structures [23,24,25,26]. Efforts to advance 3DPC applications are expanding, and the frontier of underwater 3D printing has garnered increasing research attention. Early studies have explored the development of appropriate mixtures and printing systems for underwater deployment [27,28,29,30,31], including the use of traditional and alternative anti-washout admixtures, the assessment of buildability considering fresh state strength and resistance to hydrostatic pressure [32,33,34], the assessment of the mechanical performance, and the effect of anisotropy in underwater 3D-printed materials [17,35].

This paper aims to advance the understanding of utilizing marine-derived materials in 3D printing of concrete (3DPC), with a specific focus on evaluating the impact of seawater incorporation into conventional 3DPC mixtures. The study investigates the behavior and characteristics of cementitious systems when seawater is used as mixing water, providing insights into both conventional 3D printing conducted in in-air conditions and underwater 3D printing performed in static submerged environments. These considerations include the challenges associated with maintaining the integrity of printed layers against fluid forces in submerged conditions, mitigating washout or dispersion of the cementitious mixture during printing. By comparing conventional and underwater production processes, this paper seeks to identify the critical design parameters and adjustments necessary for achieving durable and efficient underwater 3DPC systems. Ultimately, the findings from this study could provide a foundation for broader applications of 3D printing technology in marine construction, including artificial reefs, underwater foundations, coastal protection, and other sustainable infrastructure solutions. Incorporating seawater into 3DPC mixtures represents an opportunity to reduce reliance on scarce freshwater resources, reduce building costs associated with transport, and develop next-generation printing techniques tailored for challenging marine environments.

## 2. Materials and Methods

### 2.1. Materials

The materials used for the preparation of mixtures for 3D printing consisted of ASTM C595 Portland Limestone Cement (PLC 10 MS, Holcim, Chicago, IL, USA) and natural fine aggregate following ASTM C778 [36] for graded sand with a maximum particle size of 600 μm. Two types of mixing water were used, deionized freshwater (FW) and simulated seawater (SW) prepared using commercially available sea salt dissolved in deionized water to replicate the composition of ASTM D1141 substitute ocean water [37]. Three types of commercial chemical admixtures were used to control the extrudability and buildability of the 3D-printed mortars; an anti-washout admixture (AWA) (MasterMatrix UW450, Master Builders Solutions, Beachwood, OH, USA) meeting the requirements for ASTM C1882 [38], a polycarboxylate based high-range water-reducing admixture (HRWRA) (ViscoCrete 4100, Sika Co., Lyndhurst, NJ, USA) categorized as ASTM C494 Type F, and a viscosity-modifying admixture (VMA) (Stabilizer 4R, Sika Co.) according to ASTM C494 Type S [39].

### 2.2. Methods

#### 2.2.1. General Assessment of the Use of Seawater for 3D Printing Applications

The impact of substituting freshwater with seawater as mixing water in cement-based systems was initially investigated. This preliminary characterization focused on assessing the effects of seawater on the rheological properties and heat of hydration of paste mixtures, using water-to-cement (w/c) ratios commonly used in the development of concrete and 3D-printed concrete mixtures, including w/c: 0.35, 0.40, and 0.45. Furthermore, 3D printing in “in-air” conditions is well-understood, and initial development of mortar mixtures with appropriate extrudability and buildability can be achieved after a few design iterations. However, deploying cement-based mixtures in “underwater” environments introduces additional challenges to conserve the quality of individual filaments during the extrusion process and the buildability of structures; thus, the use of admixtures and modifications of the extruding system must be considered. In this case, an iterative approach was employed to incorporate simulated seawater as mixing water, starting from a baseline mixture and progressively adjusting the combination and dosage of admixtures to achieve extrudability and rheological properties comparable to mixtures prepared with deionized water. Quantitative analysis of rheological properties enabled a detailed understanding of the changes in dynamic yield stress and plastic viscosity needed for the successful integration of seawater in mortar mixtures and their application in underwater 3D printing environments. Subsequently, printability testing was conducted in an underwater environment to ensure both buildability and extrudability requirements were met.

#### 2.2.2. 3D Printing System

**Figure 1:**
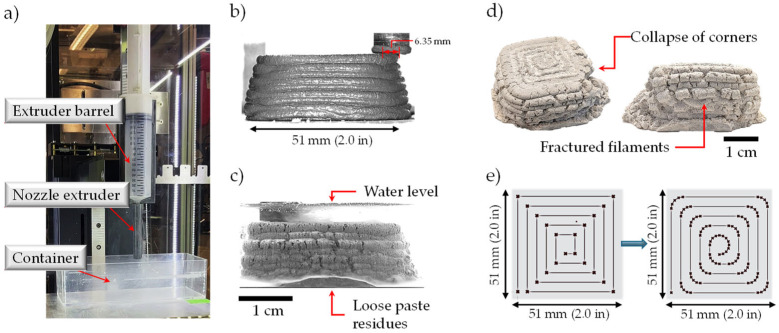
(**a**) Printing system and underwater setup; (**b**) 3D printing in air; (**c**) 3D printing in underwater conditions; (**d**) defects on initial samples 3D-printed underwater; (**e**) modification of G-code to enhance stability of 3D-printed elements.

The printing system used for the exploration of in-air and underwater 3D printing consisted of a Hyrel 30M printer, integrated with a 150 cm^3^ extruding system set up in laboratory conditions (20 °C ± 2 °C, 20% ± 5% R.H.). A 90 mm long nozzle extruder was designed to allow the placement of a prismatic container for underwater printing (Figure 1a). The inner diameter (nominal filament width) of the extruder was 6.35 mm with a nominal height of 3.0 mm; both parameters were used for the design of the toolpath. Cubes of 50 × 50 × 50 mm were designed to be used as the experimental unit to evaluate the success of the 3D printing mixtures developed (Figure 1b). While initial experiments “in air” resulted in a uniform and continuous extrusion with a stable element, it was observed that during initial underwater printing trials (Figure 1c), the element showed significant breakage of the filaments and collapse at the 90° corners due to the interaction of the filaments with the surrounding water by means of increased dispersion of the extruded material and higher slippage between filaments (Figure 1d). Thus, the flow rate was adjusted from 15 cm^3^/min to 18 cm^3^/min for the underwater condition and kept at this flow rate for the samples printed in air. Additionally, a modified printing path was prepared to smooth the change in direction of the extruder and prevent the collapse of the corners of the 3D-printed element (Figure 1e).

The development of mixtures using seawater (SW) for underwater 3D printing (U3DP) conditions were based on an iterative process based on the pronounced changes in the bleeding (displacement and separation of water from the mixture) and initial extrudability of the material, visual observations of the quality of the printed filaments, and clogging of the extruding system during the printing process. Adjustments to the chemical admixture dosages used were performed to enhance the printability and quality of the 3D-printed specimens. Table 1 shows the mixture proportioning of the successful mortar mixtures developed for 3D printing in in-air (3DP) and underwater (U3PD) conditions, using freshwater (FW) or seawater (SW) as the mixing water. These mixtures used a fixed water-to-cement (w/c) ratio of 0.32 and sand-to-cement ratio (s/c) of 0.75, by mass.

#### 2.2.3. Rheological Characterization

**Figure 2:**
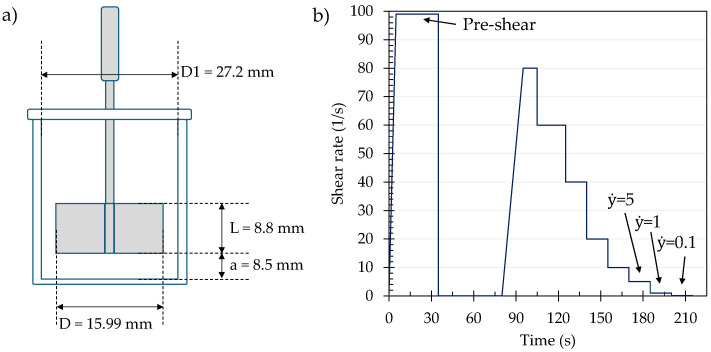
Experimental setup for rheological characterization. (**a**) Shear-vane configuration geometry; (**b**) procedure used to determine the flow curve and apparent viscosity as a function of different shear rates (ẏ) applied.

The rheological characterization of cement pastes and mortars was performed (HAAKE MARS 40, ThermoFisher, Waltham, MA, USA) with a shear-vane and cup configuration (Figure 2a). The characterization of the use of seawater as mixing water in cement-based systems was performed on pastes prepared at different water–cement ratios (w/c), whereas the characterization of the mixtures used during 3D printing was performed on the mortar obtained from the extruder before and after the printing process. This allowed the evaluation of the changes in the material as the 3D printing cubes were being fabricated. In both cases (i.e., cement and mortar materials), a sample of 50 g of cement paste was inserted into the cup and tapped to remove excess air. A pre-shear step was initially applied to the mixture to ensure homogeneity at a rate of 100 s^−1^ for 30 s, followed by a rest step of 45 s. Data collection started after an initial shear ramp to 80 s^−1^ with successive stepwise decreases with 15 s long steps to record the descending flow curve and apparent viscosity at different shear rates, following the protocol shown in Figure 2b. The dynamic yield stress and plastic viscosity were then calculated from the flow curve utilizing the Bingham plastic model [40].

#### 2.2.4. Isothermal Calorimetry

The heat evolution and cumulative heat of hydration of the mixtures was measured using a TAM Air–8-channel Isothermal Calorimeter (TA Instruments, New Castle, DE, USA). The experiments were performed at 23 °C and heat release data was collected for 100 h for all mixtures. After completion, data was then normalized by mass of cement (mW/g). Samples were prepared by mixing the corresponding water (i.e., freshwater or seawater) and cement in a vacuum mixer for 90 s. Then, approximately 15 g of paste was added to a glass ampoule, weighed, and then placed inside the calorimeter unit. Data collection started 45–90 min after the initial mixing of each sample.

#### 2.2.5. Compressive and Flexural Strength

**Figure 3:**
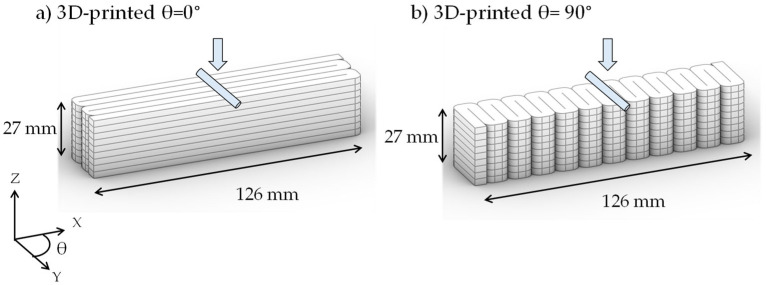
Beams for flexural strength evaluation of (**a**) filament strength (θ = 0°) and (**b**) interfacial strength (θ = 90°).

The assessment of the mechanical performance of 3D-printed elements produced with different mixtures was evaluated through compressive and flexural strength testing. Sets of cubes with nominal dimensions of 50 × 50 × 50 mm were produced for each of the proposed mixtures. The actual dimensions of the 3D-printed elements were recorded and used to accurately estimate their compressive strength. Samples were cured in limewater for 28 days before testing in a compressive strength machine with a capacity of 1112 kN (250 kips) (Gilson Company, Inc., Lewis Center, OH, USA), following the testing protocol described in ASTM C109 [41]. The assessment of flexural strength consisted of two sets of 3D-printed beam samples per mixture. The first set of beams was printed with filament orientation parallel to the length of the beam (θ = 0°), enabling the assessment of the strength of the filaments (Figure 3a). The second set of beams was printed with the filaments parallel to the width of the beam (θ = 90°), allowing for the evaluation of the interfacial strength (Figure 3b). These samples were cured in limewater for 7 days and tested in a universal testing system (Instron Company, Inc., Norwood, MA, USA) equipped with a 3-point bending fixture with a support span length of 39 mm (3 inches) and displacement rate of 0.5 mm/min.

#### 2.2.6. Dimensional Accuracy of 3D-Printed Elements

The dimensional accuracy of the 3D printing samples produced with the different mixtures in air and underwater was assessed by scanning the cross-section of the fractured beam specimens following the flexural strength tests [42]. A digital representation of the contour of the 3D-printed element was generated using Rhinoceros 3D version 8 (Robert McNeel & Associates, Seattle, WA, USA), followed by the determination of the area (*a*), centroid (*c*), and second moment of area (*I*). The contour of the cross-section was used to identify the deviations between the measured and designed geometry. Conversely, the flexural strength (*σ*) was determined using the geometric properties of the cross-section, the peak load (*F*) obtained from the 3-point bending test, and the length of the support span (*L*) as shown in Equation (1). This dual-purpose evaluation enables a better understanding of both mechanical properties and the fidelity of the 3D printing process in producing the intended geometries.

(1) $$\sigma =\frac{M\times c}{I},\;\;\;\;\;\;\;\;\;where\;\;M=\frac{F\times L}{4}$$

#### 2.2.7. Hardened-State Density

The density of the 3D-printed elements was determined by using a 50–60 g sample obtained from the specimens tested during flexural testing. Prior to measurement, the samples were dried for 8 h at 60 °C and subsequently stored in sealed plastic bags to prevent moisture absorption prior to testing. A gas pycnometer (Accupyc III, Micromeritics, Norcross, GA, USA) was utilized to measure the volume of the sample by employing the gas displacement technique, which allows the density to be determined based on its known mass. During the process, helium gas was introduced into a 100 cm^3^ chamber containing the sample and three cycles were used to determine average density to ensure accuracy and consistency. This standardized procedure allowed for reliable assessment of the printed materials’ density to assess the effect of the printing environment (i.e., in-air or underwater) and materials (i.e., use of seawater as mixing water) used in the 3D printing process.

#### 2.2.8. Statistical Analysis

The error bars in reported figures represent ± one standard deviation of the average of the samples tested. To determine significance between the averages of two sets of samples, ANOVA testing followed by Tukey–Kramer HSD analysis was performed. When reported, *p*-values were calculated via Tukey–Kramer HSD analysis at the 95% confidence level, with a *p*-value < 0.05 indicating significance.

## 3. Results

### 3.1. Assessment of the Effect of Seawater on Rheology and Heat of Hydration

**Figure 4:**
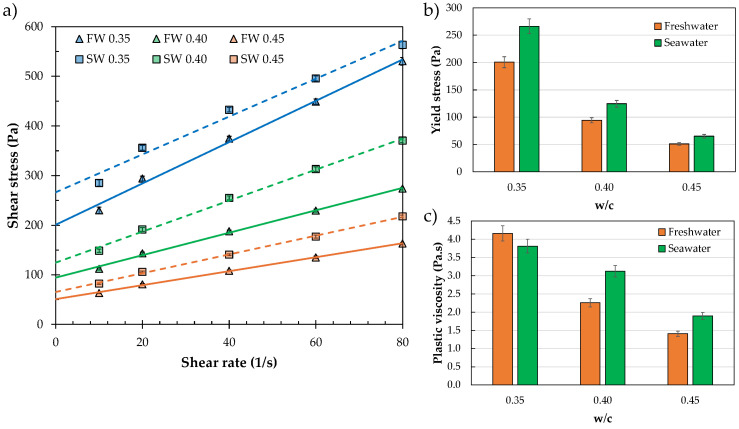
Effect of using freshwater (FW) and seawater (SW) on the rheological properties of cement paste systems. (**a**) Data fitted to Bingham model; (**b**) yield stress evolution with increasing w/c; (**c**) plastic viscosity evolution with increasing w/c.

The rheological evaluation of mixtures containing seawater as a 100% replacement for freshwater revealed significant changes in the resulting flow curve (Figure 4a). The Bingham model was used to determine the static yield stress and plastic viscosity as the primary rheological parameters. It was observed that seawater increased the yield stress by an average of 31% across the different w/c ratios evaluated (Figure 4b). For w/c ratios of 0.40 and 0.45, the measured plastic viscosity increased when seawater was used. However, at a lower w/c (i.e., 0.35), typical of 3D printing mixtures, a slightly lower viscosity was observed (Figure 4c). These results provide valuable insights into the mixture design process, guiding the dosage of admixtures (e.g., viscosity modifiers and water-reducing admixtures) needed to achieve optimal printability in both “in-air” and “underwater” conditions.

**Figure 5:**
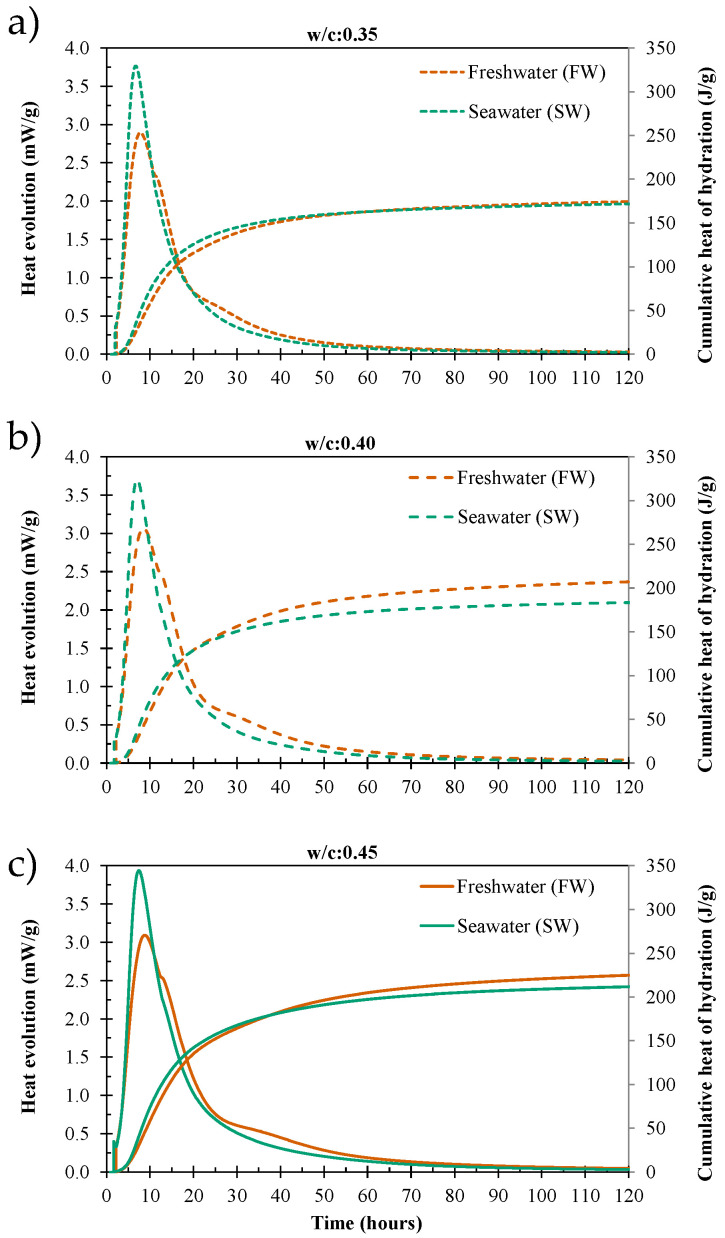
Isothermal calorimetry performance of cement-paste mixtures using freshwater (FW) and seawater (SW). (**a**) w/c: 0.35; (**b**) w/c: 0.40; (**c**) w/c: 0.45.

The results of the heat of hydration tests illustrate the effect of seawater on the hydration process. The isothermal calorimetry curves shown in Figure 5 highlight the general impact of seawater (SW) on heat evolution and hydration of the cement paste compared to freshwater (FW) as mixing water, notably an increase in early heat generation. This acceleration is attributed to the high concentration of dissolved salts in seawater, particularly chlorides (Cl^−^), sulfates (SO_4_^2−^), and cations such as sodium (Na^+^) and magnesium (Mg^2+^), which enhance the dissolution kinetics of tricalcium silicate (C_3_S) and tricalcium aluminate (C_3_A). The chloride ions act as accelerators, promoting faster formation of calcium silicate hydrate (C-S-H) gel and ettringite, depleting the system of sulfates, which causes the merging of the sulfate peak with the main silicate peak observed between 5 and 15 h, while the elevated ionic strength shifts equilibrium conditions and increases the overall reaction rate. However, the accelerated initial hydration is followed by the slowdown in the hydration process for the sample with w/c: 0.35, the total heat of hydration aligns after 48 h, and there is little distinction between freshwater and seawater samples until the end of the test (Figure 5a). For a w/c: 0.40, the crossover point where FW total heat is higher than the SW sample occurs after 20 h (Figure 5b), whereas for samples with w/c: 0.45, this crossover is observed after 36 h (Figure 5c). These results provide a baseline for further mixture development. This observation aligns with existing research on seawater and sea-sand concrete, which indicates that such concrete exhibits higher early-age strength (due to greater rates of hydration) but typically hinders long-term strength evolution [14,43,44].

### 3.2. Results of Rheological Characterization

The design and assessment of mixtures for underwater printing using a syringe-based system relies on rheological characterization of the paste fraction and printability tests of mortars to compare the performance of seawater- and freshwater-based mixtures in both “in-air” and “underwater” environments. Rheological characterization is performed on cement paste samples 5 min after initial mixing by determining the dynamic yield stress and plastic viscosity from the Bingham model fit to the descending stage of the flow curve.

To assess the effect of seawater on printability and its comparison to freshwater, an iterative design approach examined the influence of chemical admixtures—high-range water-reducing admixture (HRWRA), viscosity-modifying admixture (VMA), and anti-washout admixture (AWA)—on yield stress and viscosity both independently and in combination. To isolate the effect of seawater substitution, admixture dosages were kept constant relative to freshwater mixtures.

**Figure 6:**
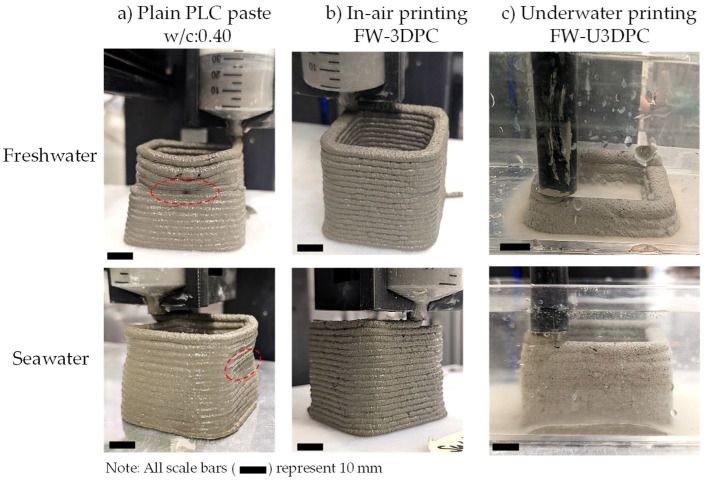
Extrudability and stability tests of 50 × 50 mm hollow elements of (**a**) plain cement paste mixtures (w/c = 0.40); (**b**) 3D-printed samples in air (FW-3DPC); (**c**) 3D-printed samples underwater (FW-U3DPC).

**Figure 7:**
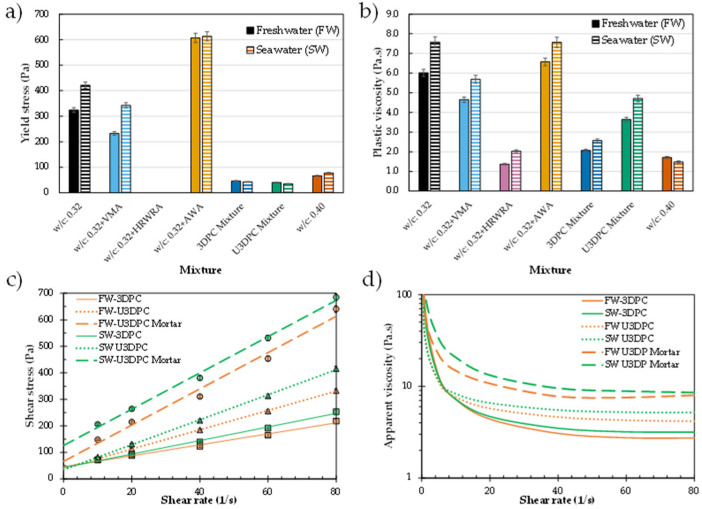
Experimental results of rheological characterization. (**a**) Yield stress and (**b**) viscosity of paste mixtures including different chemical admixtures and resulting mixture with potential for 3D printing; (**c**) comparison of flow curves and (**d**) apparent viscosity of paste and mortar mixtures for printing in in-air and underwater conditions.

The design of printable mixtures for a 6 mm nozzle extruder requires balancing two competing requirements: extrudability and buildability. A mixture of plain cement with w/c = 0.4 achieves consistent and complete extrudability, with material flowing smoothly through the nozzle. However, excessive water content results in poor buildability, as buckling and collapse occur after just a few layers (Figure 6a). Conversely, a mixture of plain cement with w/c = 0.32 produces stable, well-defined filaments and appropriate shape retention, but its high yield stress and viscosity induce bleeding and nozzle clogging during the early stages of extrusion. The clear differences between both mixtures is illustrated in Figure 7a,b, where high yield stress, although beneficial for buildability, and viscosity impair the extrudability of mortar mixtures, whereas low values of these parameters enable extrusion but compromise buildability due to excessive water content and deformation during printing.

To achieve satisfactory printability, the combined use of HRWRA and VMA was explored for “in-air” 3D printing, following demonstrated approaches for small-scale mortar 3D printing [42,45]. The HRWRA significantly reduces yield stress, enabling material flow even at low w/c ratios, while the VMA prevents bleeding during early-stage extrusion and enhances buildability. The addition of AWA further increases yield stress with minimal impact on viscosity, improving shape retention for underwater applications. The individual effect of these admixtures in a control w/c = 0.32 mixture is presented in Figure 7a,b. It is observed that yield stress and viscosity are drastically impacted by the addition of each of these admixtures at the dosage levels proposed in Table 1. It is worth noting that HRWRA addition drastically reduces the yield stress to near-zero values, effectively transforming the mixture from a yield-stress fluid exhibiting Bingham plastic behavior into a fluid with predominantly Newtonian flow characteristics. However, when HRWRA and VMA are combined to produce printable mixtures for “in-air” (3DPC) printing, the resulting materials exhibited low yield stress and intermediate viscosity values (between those of w/c = 0.32 and 0.40 mixtures), enabling smooth and complete extrusion. Printed elements using freshwater and seawater are presented in Figure 6b, illustrating continuous filaments with consistent layer quality.

A detailed comparison of the flow curve and apparent viscosity of seawater- and freshwater-based 3D-printable mixtures is presented in Figure 7c,d. For paste mixtures, seawater substitution had minimal impact on yield stress compared to freshwater counterparts, with values remaining consistent (36–46 Pa) for both in-air and underwater formulations. In contrast, plastic viscosity consistently increased with seawater substitution and was further elevated by AWA addition for underwater environments. Thus, a clear influence of the use of seawater at early ages is observed in the fresh state of paste and mortar mixtures, associated with the effect of chlorides in the rapid dissolution and reaction of silicate and aluminate phases.

To assess the translation from paste to mortar rheology, additional experiments were conducted on mortar mixtures for underwater conditions. The comparison between paste and mortar samples clearly demonstrates the substantial increase in both viscosity and yield stress contributed by sand addition, providing critical insight into the actual rheological characteristics of the materials used in subsequent 3D printing experiments.

This rheological characterization provides critical benchmark ranges for yield stress and plastic viscosity that enable smooth and continuous printing at the tested scale. These findings, particularly regarding the effects of chemical admixtures and seawater substitution, can be extrapolated to guide mixture design for larger printing scales and nozzle diameters (from centimeter- to meter-scale filament width).

### 3.3. Printability in Underwater Conditions

**Figure 8:**
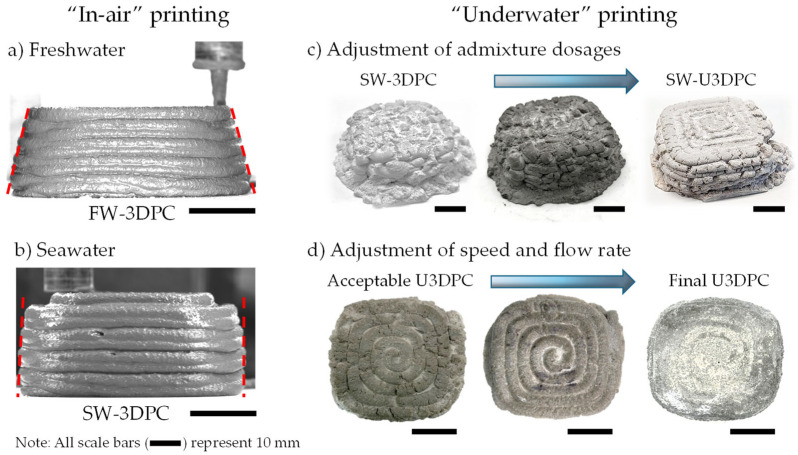
Iterative design of mixtures and element quality refinement. (**a**,**b**) Effect of using seawater as replacement of freshwater in mortar mixtures; (**c**) Effect of chemical admixture adjustment for printability underwater; (**d**) Fine-tuning of printing parameters for underwater printing. All scale bars represent 1.0 cm (0.4 inch).

To assess the mechanical performance of 3D-printed elements produced in an underwater environment, cubes and beams were prepared. Mixtures prepared with freshwater (FW-U3DPC) were printed in a freshwater environment, whereas mixtures prepared with seawater (SW-U3DPC) were printed in seawater. Initial elements produced in air demonstrated that replacing the freshwater with seawater—maintaining the same dosages of admixtures—produced a more stable 3D-printed element with lower lateral and vertical deformations (Figure 8a,b). On the other hand, the deployment of “underwater” mixtures followed a two-stage iterative process. In first place, the addition of AWA was used to guarantee that the extruded filaments were deposited continuously in the printing bed. However, this implied that a balance with the other admixtures was needed to guarantee similar viscosity and yield stress for both extrudability and buildability reasons, respectively. During this stage, it was observed that the extruded filaments were successfully deposited, yet the filaments tore down along the straight and curve segments without compromising the overall buildability of the element (Figure 8c). To enhance the quality of the 3D-printed element, the printing speed was reduced by 33% (from 900 mm/min to 600 mm/min); this strategy allows for a more stable extrusion, avoiding the dragging of the filaments usually observed in “in-air” conditions that, in underwater conditions, causes filament break and higher losses of binder. Figure 8d illustrates the effects of adjusting the extrusion rate to fit an appropriate printing speed to produce high-quality materials in underwater conditions.

### 3.4. Mechanical Performance of In-Air vs. Underwater Samples

**Figure 9:**
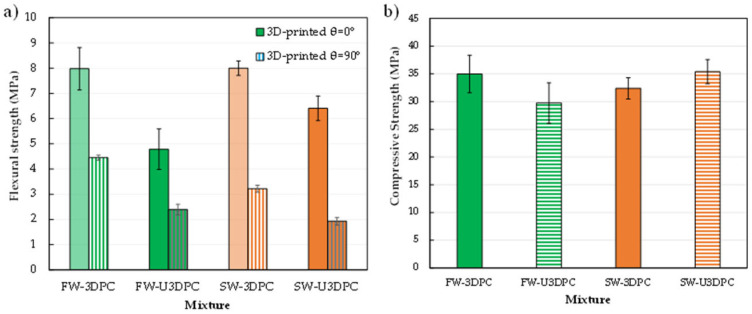
Mechanical performance of 3D printing in in-air (3DPC) and underwater (U3DPC) elements. (**a**) Flexural strength; (**b**) compressive strength.

The mechanical performance of 3D-printed mixtures including compressive and flexure strength tests are shown in Figure 9. Flexural strength results indicate a clear detrimental effect on the performance of the 3D-printed θ = 90 samples (interfacial strength) with respect to 3D-printed θ = 0° (filament strength) samples as shown in Figure 9a.

The assessment of samples 3D-printed in air shows comparable flexural strength for freshwater (FW-3DPC) and seawater (SW-3DPC) mixtures in the θ = 0° filament orientation. However, seawater samples exhibited reduced interfacial (θ = 90°) strength relative to freshwater samples.

In the case of underwater printing, samples using seawater (SW-U3DPC) showed higher filament strength (θ = 0°) compared to freshwater samples (FW-U3DPC); interfacial strength (θ = 90°) of the SW-U3DPC samples is still the lowest of the different mixtures studied and represents only 30% of the strength of the same material tested along the filament length. Nonetheless, interfacial strength (90°) was significantly lower for all underwater mixtures, representing only 30% of the corresponding filament strength. In the best scenarios (FW-3DPC and FW-U3DPC), interfacial strength was 55.8% and 50.0% of the filament strength, respectively. These findings underscore the need for further investigation into curing strategies and layer adhesion optimization, particularly in underwater printing conditions.

The results of compressive strength are presented in Figure 9b. Statistical analyses using ANOVA followed by Tukey–Kramer HSD at a 95% confidence level reveal that compressive strength between mixtures produced in in-air and in underwater conditions is not significantly different. Still, it is worth noting that FW-U3DPC samples exhibited the lowest average compressive strength (29.73 MPa), while SW-U3DPC achieved strength comparable (35.38 MPa) to FW-3DPC (34.95 MPa) and slightly higher than SW-3DPC (32.39 MPa).

These results indicate that printing methodology and interfacial bonding have a greater impact on flexural strength than on compressive strength. Further refinement of printing parameters is needed to minimize interfacial performance disparities. The mechanical performance of 3D-printed materials is influenced by printing medium, orientation, and curing conditions. Flexural strength tests highlight challenges in interfacial quality, especially for underwater printing, while compressive strength shows less pronounced variability among mixtures.

### 3.5. Hardened-State Density and Dimensional Accuracy

**Figure 10:**
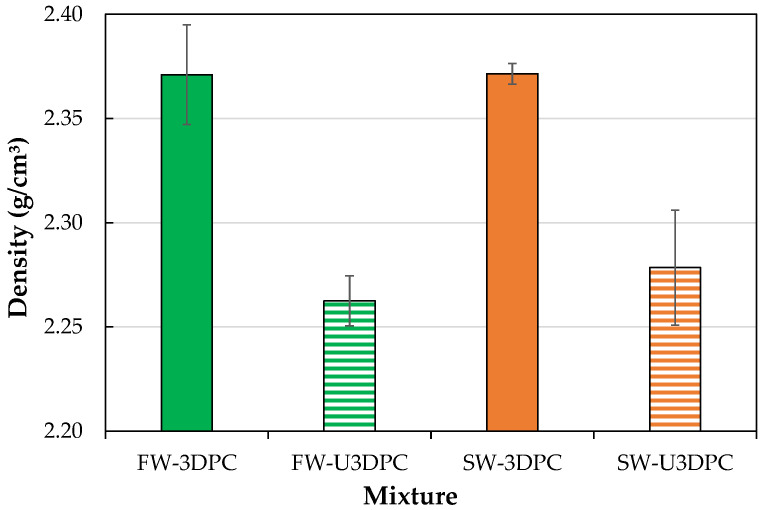
Results of density evaluation of mixtures printed in air and underwater.

Figure 10 illustrates the density of 3D-printed beams produced in air and underwater. The results indicate a decrease in density for samples printed underwater. On average, density decreased 4.6% for beams printed in freshwater and 3.9% for those printed in seawater, while the type of water (freshwater or seawater) did not influence the measured density. These results can be analyzed based on the technique used for density measurement. By using a gas pycnometer, density measurements excluded accessible pores, thus lower density indicates higher internal porosity in underwater-printed samples, which could be initially attributed to the interaction between freshly deposited filaments and the surrounding water during the extrusion, possibly affecting the microstructure of the material, not perceptible in the bulk condition but with great incidence in the mechanical performance of underwater elements.

The assessment of dimensional accuracy in 3D-printed samples is critical for determining their geometric properties, which are essential for the estimation of flexural strength. Additionally, evaluating the cross-section of 3D-printed elements allows for comparison between the actual cross-section and the ideal designed beam cross-section under the assumption of consistent cross-sectional geometry along the length of the element.

**Figure 11:**
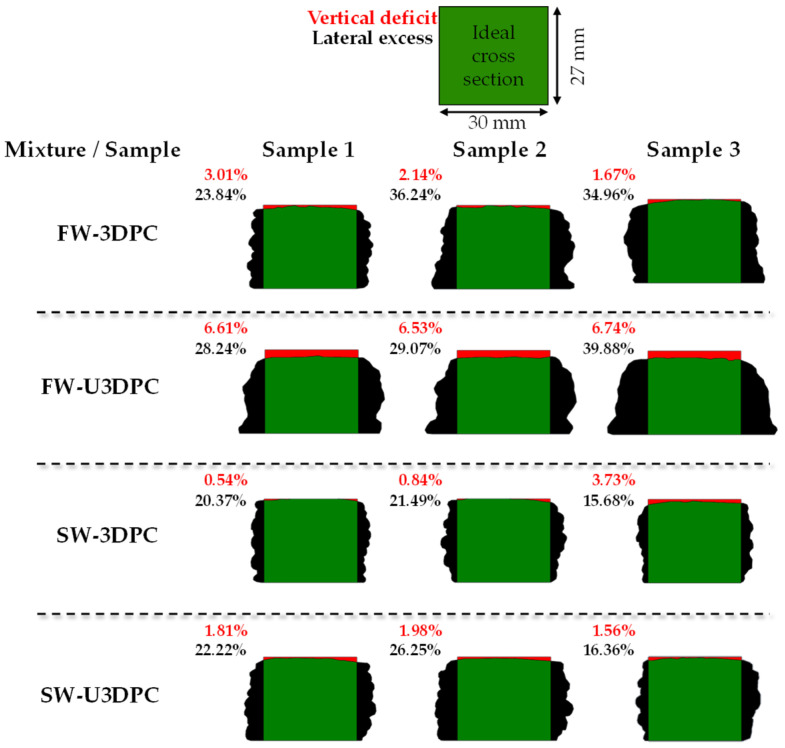
Cross-section of different samples used to determine dimensional accuracy of 3D-printed samples illustrating the vertical deficit (red) and lateral excess (black) of the material with respect to the ideal cross-section of the designed geometry.

Two indexes are used to characterize the quality of the 3D-printed elements: “vertical deficit”, which represents the overall settling that occurs after all layers are deposited, and “lateral excess,” which reflects the degree of material spreading during the printing process. The cross-sectional analysis of 3D-printed samples in in-air and underwater conditions is presented in Figure 11. It was observed that samples printed in air with freshwater (FW-3DPC) exhibit slightly higher vertical and lateral deformation compared to samples printed with seawater (SW-3DPC). Furthermore, underwater samples printed with freshwater demonstrate larger deformation compared to those printed with seawater (SW-U3DPC), which demonstrates a comparable quality with respect to samples printed in air. These deformations may help explain the density differences observed in Figure 10. Given that identical printing parameters were used across samples, a constant flow and volume of material was deposited, indicating that a larger cross-section corresponds to disturbances produced by the presence of water, leading to higher internal porosity and the impact on the quality of the filament interfaces, inherently affecting the mechanical performance of the samples.

## 4. Considerations of Underwater 3D Printing of Concrete

The growing interest in deploying concrete structures directly underwater necessitates the convergence of three critical domains: cementitious material development, extrusion system design with monitoring controls, and comprehensive understanding of the deployment environment where marine infrastructure will operate.

Material optimization for underwater environments must ensure adequate rheological performance, cohesion, and resistance to washout while maintaining compatibility with both seawater and freshwater environments. The use of seawater as mixing water introduces additional considerations, particularly regarding chloride intrusion and its potential limitations for certain applications. Addressing these durability concerns through appropriate chemical admixtures, supplementary cementitious materials, or protective measures is essential for long-term structural performance. However, seawater used as mixing water stiffens the fresh mixture and accelerates strength gain, which are both beneficial for underwater printing.

Structural performance challenges identified in this study illustrate the consistently reduced interfacial strength observed in underwater printing, which may compromise structural integrity. Enhancing interlayer bonding quality and minimizing defects during layer deposition through optimized curing strategies and refined printing parameters are critical priorities for improving mechanical performance and ensuring structural reliability.

Despite these challenges, underwater 3D printing of concrete (3DPC) holds significant promise for marine infrastructure projects. However, successful implementation requires design strategies that balance structural requirements with the practical constraints of deployment and operation in submerged conditions, including dynamic loading scenarios and environmental variability. Bridging the gap between controlled laboratory conditions and the complex realities of underwater construction environments remains the critical challenge for advancing underwater 3D printing technology toward practical implementation. Thus, future research should prioritize several key areas, including exploration of advanced binder systems and non-traditional reinforcement strategies to improve underwater printing performance; development of chemical admixtures specifically tailored for submerged construction; and real-world validation in controlled marine test sites to refine processes and demonstrate scalability for diverse underwater applications. Most importantly, consideration of external factors that influence printing equipment deployment and consequent print quality, such as water currents, visibility limitations, positioning accuracy, and material transport logistics, is vital for translating lab-scale demonstrations into reliable full-scale field infrastructure.

## 5. Conclusions

This investigation of rheological properties and mechanical performance of 3D-printed cementitious materials for underwater applications using seawater as mixing water provides the following key findings:

1. The successful formulation of printable mixtures for both in-air and underwater environments requires a balance between extrudability and buildability. For this purpose, rheological characterization established the appropriate dynamic yield stress and viscosity to enable smooth extrusion while maintaining adequate shape retention in a syringe-based printing system with the combined use of chemical admixtures, preventing bleeding, enhancing buildability, and improving shape retention in underwater conditions.
2. Seawater substitution demonstrated minimal impact on yield stress but consistently increased plastic viscosity compared to freshwater mixtures. This viscosity increase contributed to improved dimensional stability without compromising the extrudability. The observed increase in hydration coupled with the increased viscosity are both beneficial to the underwater 3D printing environment.
3. Mechanical testing revealed significant anisotropy in 3D-printed elements, with interfacial strength substantially lower than filament strength. In the case of compressive strength, results showed less sensitivity to printing environment and water type, with values ranging from 29.73 to 35.38 MPa at 28 days and no statistical difference between in-air and underwater samples, highlighting the potential of using seawater in 3D printing of concrete applications.
4. Density measurements revealed 3.9–4.6% lower density in underwater-printed samples compared to in-air counterparts, independent of water type (freshwater or seawater). This reduction may indicate higher internal porosity resulting from water interaction during filament deposition. The increased internal porosity and compromised interfacial bonding in underwater conditions directly correlate with reduced flexural strength, highlighting the critical role of layer adhesion quality in mechanical performance.

Further investigation to advance underwater 3D printing technology includes microstructural optimization to minimize internal porosity and enhance interfacial strength, which can lead to a refined experimental and model-based assessment of seawater-mixed 3D-printed concrete performance in different marine environments (pH, salinity, contaminants, etc.), including chloride ingress, corrosion resistance, and bond strength evolution over time.

## Figures and Tables

**Table 1:** Mixture proportioning of mortar mixtures for in-air and underwater 3D printing.

Mixture	w/c	s/c	Cement	Sand	Water	HRWRA	VMA	AWA
			kg/m^3^	kg/m^3^	kg/m^3^	mL/100 kg	mL/100 kg	mL/100 kg
FW-3DP	0.32	0.75	1036.3	777.2	339.4	925	940	0
FW-U3DP	0.32	0.75	1036.2	777.1	339.3	925	470	525
SW-3DP	0.32	0.75	1036.3	777.2	339.4	925	940	0
SW-U3DP	0.32	0.75	1036.1	777.1	339.3	1110	470	525

## Data Availability

The original contributions presented in this study are included in the article. Further inquiries can be directed to the corresponding author.
